# Thermal Stability and Phase Evolution in the Phosphorus-Containing High-Entropy Alloy Fe_22_Ni_16_Co_19_Mn_12_Cr_16_P_15_

**DOI:** 10.3390/ma18235261

**Published:** 2025-11-21

**Authors:** Krzysztof Ziewiec, Marcin Jasiński, Aneta Ziewiec

**Affiliations:** 1 Institute of Technology, University of the National Education Commission (UKEN), ul. Podchorążych 2, 30-084 Krakow, Poland; marcin.jasinski@uken.krakow.pl; 2 AGH University of Krakow, Faculty of Metals Engineering and Industrial Computer Science, al. A. Mickiewicza 30, 30-059 Krakow, Poland; aziewiec@agh.edu.pl

**Keywords:** high-entropy alloys, Fe–Ni–Co–Mn–Cr–P alloy, eutectic crystallization, amorphous ribbons, DSC, SEM/EDS, XRD, thermal stability, phosphides

## Abstract

This study investigates the Fe_22_Ni_16_Co_19_Mn_12_Cr_16_P_15_ alloy designed to enhance glass-forming ability. The alloy was synthesized by arc melting and examined using infrared thermography, differential scanning calorimetry (DSC), scanning electron microscopy with energy-dispersive spectroscopy (SEM/EDS), and X-ray diffraction (XRD). Thermographic measurements revealed a temperature arrest at ~1007 K associated with eutectic crystallization, accompanied by contraction visible as a flattened ingot surface. DSC confirmed the dominant eutectic transformation (−170.7 J/g). Compared with the previously studied Fe_22_Ni_16_Co_19_Mn_12_Cr_16_P_15_ alloy, this composition showed a simplified transformation sequence and a larger eutectic fraction. DSC of melt-spun ribbons demonstrated a three-step crystallization (659 K, 699 K, 735–773 K, completion ~820 K) with a total enthalpy of 180.4 J/g. The broad crystallization interval (ΔTc ≈ 161 K) indicates enhanced thermal stability compared with simpler Ni–P or Fe–Ni–P–C alloys. SEM/EDS observations revealed eutectic colonies with predominantly rod-like morphology and chemical partitioning in inter-colony regions, favoring precipitation of transition metal phosphides. XRD confirmed four crystalline phases (Fe–Ni, CrCoP, Ni_3_P, MnNiP) in ingots, while ribbons exhibited a fully amorphous structure. These findings demonstrate that Fe_22_Ni_16_Co_19_Mn_12_Cr_16_P_15_ possesses good glass-forming ability but forms multiple phosphides under slower cooling. Precise cooling control is thus essential for tailoring its amorphous or crystalline state.

## 1. Introduction

High-entropy alloys (HEAs) and compositionally complex alloys have recently attracted considerable attention due to their unique physical and chemical properties. These properties arise from high configurational entropy, sluggish diffusion, and the ability to stabilize both solid solutions and intermetallic compounds. A well-known example is the classical CoCrFeMnNi alloy, where a stable FCC solid solution is retained despite significant differences in the free energies of the constituent elements [1,2].

An increasing number of studies indicate that non-metallic elements such as phosphorus, boron, and silicon play an important role in determining the microstructural stability of HEAs. These elements enhance the glass-forming ability but simultaneously promote the formation of stable phosphides, borides, or silicides under slower cooling conditions, leading to complex phase constitutions [3,4].

Phosphorus plays a particularly dual role. On the one hand, it reduces crystallization temperatures and extends the supercooled liquid region, which facilitates amorphization. On the other hand, it easily segregates and triggers the precipitation of phosphides, thereby complicating the crystalline structure and leading to the presence of multiple intermetallic phases instead of a simple solid solution [5,6]. Similar behavior has been observed in alloys with boron, where stable borides form [7,8,9], and in alloys with silicon, where silicides are present [4,10,11].

In phosphorus-containing alloys, the balance between retaining the amorphous state and the tendency to undergo multiphase crystallization during cooling is of particular importance. DSC studies of Ni–P or Pd–Ni–P alloys demonstrate that rapid cooling favors amorphization, while reheating reveals multi-step crystallization with phosphide formation [12,13]. This balance between thermal stability of the glassy state and the tendency toward multiple crystalline phases is key for interpreting the present results.

Recent studies indicate that adding phosphorus to Fe-, Ni-, and Co-based systems can enhance glass-forming ability, while slow cooling promotes segregation and the precipitation of transition metal phosphides, leading to complex multiphase constitutions [3]. Comparable multi-stage crystallization behavior has been reported in P-bearing metallic glasses and ribbons (e.g., Ni–P and Pd–Ni–P), where rapid quenching favors amorphization but reheating triggers successive crystallization events of phosphide phases [5,6,12,13]. In high-entropy or compositionally complex alloys, this dual action of P—stabilizing the glassy state yet facilitating phosphide formation under near-equilibrium conditions—remains a key design constraint for P-rich systems.

Similar solidification-analysis methodologies have also been applied in other alloy systems, for example, in recent studies on 316L stainless steels using advanced in situ techniques [14,15]. Although these materials differ fundamentally from the phosphorus-containing high-entropy alloy examined here, these works illustrate related methodological approaches, while the present methodology was adapted to the specific characteristics of P-containing HEAs.

Building on our previous study of the Cr_16_Mn_16_Fe_16_Co_16_Ni_16_P_20_ alloy [16], where solidification produced several primary (non-eutectic) phases and resulted in a complex multi-step crystallization sequence, the present work examines a modified Fe_22_Ni_16_Co_19_Mn_12_Cr_16_P_15_ composition. The adjustments—higher Fe and Co, lower Mn, and a reduction in P from 20 to 15 at.%—were introduced to suppress primary solidification and shift the alloy toward conditions favoring cooperative eutectic growth. Studies of eutectic alloy systems capable of forming metallic glasses under rapid quenching show that glass-forming ability is strongly influenced by the configuration of the eutectic coupled zone: coupled eutectic growth requires continuous solute partitioning ahead of the growth front, which imposes significant kinetic constraints and reduces the probability of primary crystallization at high cooling rates, whereas primary solid solution phases can grow in a nearly partition less manner, enabling rapid massive crystallization that strongly suppresses vitrification [17,18,19]. Thermodynamic analyses of equilibrium versus partition less solidification further demonstrate that when the liquid approaches the T_0_ boundary, diffusion less crystallization is favored, and avoiding primary solidification becomes essential for improving glass-forming ability [20,21].

The present compositional modification is therefore motivated by both (i) experimental evidence from our earlier P-containing HEA, where extensive primary crystallization limited the role of eutectic reactions [16], and (ii) established mechanistic criteria indicating that suppressing primary phases and strengthening eutectic-controlled solidification enhances the ability of multicomponent alloys to bypass crystallization during rapid cooling. This provides a clear and well-supported rationale for analyzing the crystallization behavior and thermal stability of the Fe_22_Ni_16_Co_19_Mn_12_Cr_16_P_15_ alloy.

## 2. Materials and Methods

Alloy Preparation. The Fe_22_Ni_16_Co_19_Mn_12_Cr_16_P_15_ alloy was synthesized by arc melting high-purity elemental powders (≥99.9 wt.%). Melting was carried out in a water-cooled copper crucible under a protective atmosphere of high-purity argon (99.999%). To improve chemical homogeneity, each ingot (20–30 g) was re-melted at least six times, being flipped between cycles. Part of the alloy was subsequently re-melted and rapidly solidified into ribbons by the melt-spinning method on a copper wheel rotating at a linear speed of 40 m/s, yielding ribbons of about 20 μm in thickness.

Thermographic Analysis. The cooling process of arc-melted ingots was monitored using a FLIR SC7650 mid-wave infrared camera (FLIR Systems, Wilsonville, OR, USA). Thermal data were collected through a CaF_2_ window and processed using the ResearchIR 4.40 and Altair software. The temperature evolution was analyzed in a defined region of interest (ROI), allowing determination of cooling rates and identification of thermal arrests associated with solidification reactions.

The cooling process of arc-melted ingots was monitored using a FLIR SC7650 short-wave infrared camera (1–3 µm spectral range, InSb detector, FLIR Systems, Wilsonville, OR, USA). The thermal sensitivity (NETD) of the system is below 20 mK. The analysis was based on the maximum apparent surface temperature within a defined region of interest (ROI), as this parameter is most sensitive to heat release during phase transformations. The measured temperatures are radiometric and were used to identify the onset and course of the eutectic transformation rather than to determine absolute melting temperatures.

Differential Scanning Calorimetry (DSC). Thermal analysis was performed using a Netzsch STA 449 calorimeter (NETZSCH-Gerätebau GmbH, Selb, Germany) under a flowing argon atmosphere (99.999%). Measurements were conducted for both ingots and melt-spun ribbons at a constant heating/cooling rate of 20 K/min. The enthalpies of thermal effects were calculated from the peak areas of the DSC curves.

Microstructural Characterization. The microstructure of ingots was analyzed using a JEOL JSM scanning electron microscope (JEOL Ltd., Akishima, Tokyo, Japan) equipped with an energy-dispersive X-ray spectrometer (EDS). SEM observations were performed on mechanically polished, unetched cross-sections using backscattered electron (BSE) imaging to reveal compositional contrast between eutectic phases. Both secondary electron (SE) and backscattered electron (BSE) modes were applied to examine morphology, while EDS elemental mapping was used to investigate chemical partitioning.

X-ray Diffraction (XRD). Phase analysis of both ingots and melt-spun ribbons was performed on a Rigaku SmartLab diffractometer (Rigaku Corporation, Akishima, Tokyo, Japan) using Cu Kα radiation (λ = 1.5406 Å). The scans were collected in the 2θ range of 10–120° with a step size of 0.02°. Phase identification was carried out by comparison with PDF-5+ reference database cards.

## 3. Results 

### 3.1. Thermographic Analysis

**Figure 1:**
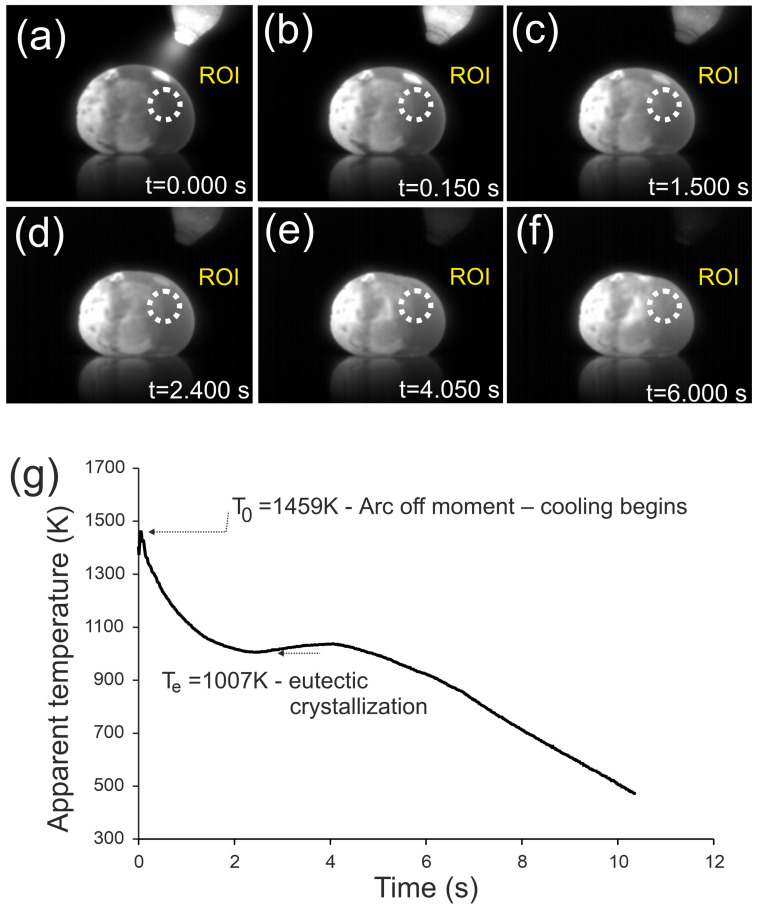
Infrared images of the Fe_22_Ni_16_Co_19_Mn_12_Cr_16_P_15_ alloy ingot after arc extinction, recorded at different times during cooling: (**a**) t = 0.0 s, (**b**) t = 0.15 s, (**c**) t = 1.5 s, (**d**) t = 2.4 s, (**e**) t = 4.05 s, (**f**) t = 6.0 s. Arrows indicate the flattened surface observed as a result of eutectic crystallization. (**g**) Temperature–time curve recorded in the ROI.

This subsection presents infrared thermography during ingot cooling and the thermal arrest associated with eutectic crystallization (Figure 1).

Figure 1 presents the cooling process of the Fe_22_Ni_16_Co_19_Mn_12_Cr_16_P_15_ ingot after arc extinction, recorded by infrared thermography. The initial temperature was T_0_ = 1459 K. The temperature–time curve shows a rapid cooling stage down to ~1007 K within 2.1 s, a plateau corresponding to eutectic crystallization lasting about 2 s, and further cooling down to 500 K within 5.9 s. The average cooling rate over the entire range was 95.9 K/s, with a maximum of 215.2 K/s before eutectic crystallization and 85.9 K/s afterwards.

In addition to the thermal arrest, distinct morphological changes were observed. At t = 1.5 s the upper surface of the ingot remained convex, similar to earlier stages, while at t = 2.4 s a slight reduction in curvature appeared. At t = 4.05 s, a clear contraction was visible, manifested as a flattened region on the upper right surface, which persisted at t = 6.0 s. This effect is directly associated with eutectic crystallization and reflects volume shrinkage during the transformation.

### 3.2. Differential Scanning Calorimetry (DSC)

**Figure 2:**
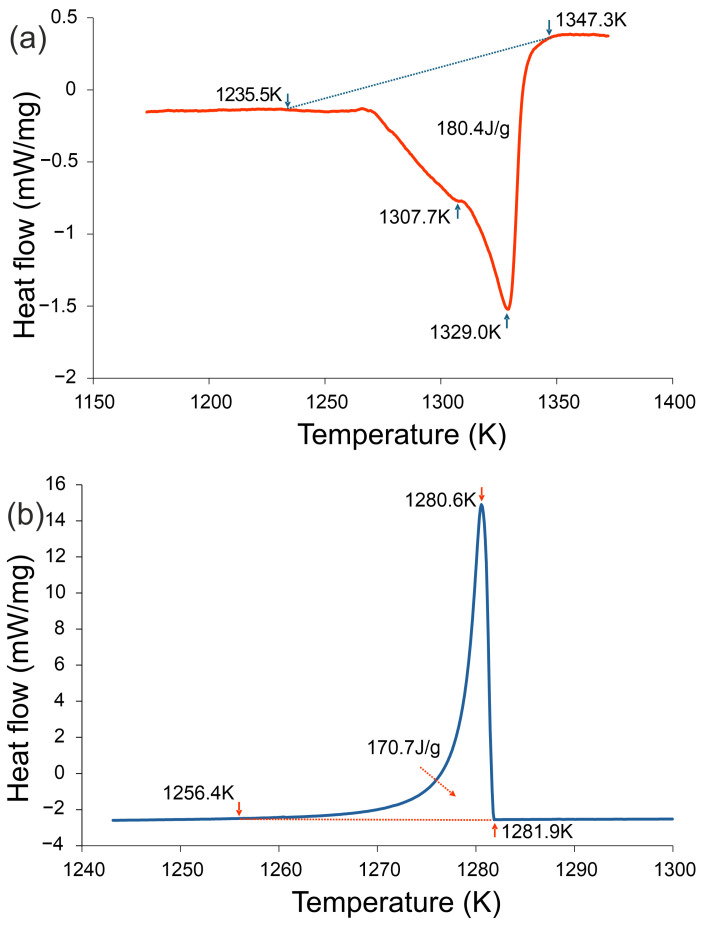
DSC curves of the Fe_22_Ni_16_Co_19_Mn_12_Cr_16_P_15_ alloy recorded at a heating/cooling rate of 20 K·min^−1^: (**a**) heating curve and (**b**) cooling curve. During heating, two endothermic effects were detected, the main one corresponding to melting (1235.5–1347.3 K, enthalpy 180.4 J·g^−1^). During cooling, a strong exothermic effect appeared in the range 1256.4–1281.9 K, associated with eutectic crystallization (enthalpy −170.7 J·g^−1^).

Here we analyze heating/cooling DSC, identify melting and eutectic ranges, and relate them to the thermography (Figure 2).

Differential Scanning Calorimetry (DSC). Figure 2 presents the DSC traces recorded during heating and cooling at 20 K/min. During heating, two distinct endothermic effects were detected, the main one corresponding to melting (1235.5–1347.3 K, enthalpy 180.4 J/g). On cooling, a strong exothermic effect was observed in the range 1281.9 K–1256.4 K, associated with eutectic crystallization (enthalpy −170.7 J/g). These results are consistent with the thermographic observations (Figure 1), where a temperature plateau at ~1007 K indicated eutectic transformation. Both thermal analyses confirm that crystallization in the investigated alloy occurs at significantly reduced temperatures compared to the melting onset, highlighting its enhanced glass-forming tendency.

### 3.3. Microstructural Characterization (SEM/EDS)

**Figure 3:**
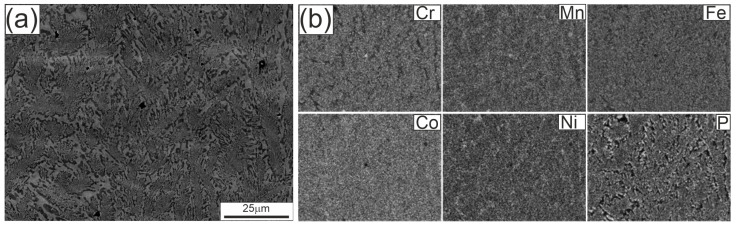
Microstructure of the Fe_22_Ni_16_Co_19_Mn_12_Cr_16_P_15_ alloy ingot after arc remelting on a copper plate: (**a**) SEM image at lower magnification, (**b**) elemental distribution maps of Cr, Mn, Fe, Co, Ni, and P obtained by EDS. Panel (**a**) was obtained in BSE mode on a polished, unetched cross-section; the compositional contrast corresponds to Fe–Ni–Mn-rich solid solution (bright) and P/Cr-rich phosphides (dark).

**Figure 4:**
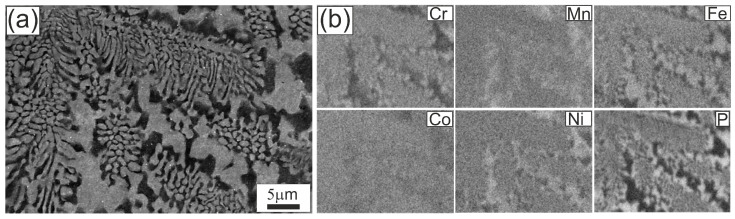
Microstructure of the Fe_22_Ni_16_Co_19_Mn_12_Cr_16_P_15_ alloy after arc remelting on a copper plate: (**a**) SEM image at higher magnification showing the rod-like eutectic morphology; (**b**) EDS elemental distribution maps for Cr, Mn, Fe, Co, Ni and P.

We show eutectic colonies, rod-like morphology, and elemental partitioning supported by EDS mapping (Figure 3 and Figure 4).

Figure 3 shows the microstructure of the Fe_22_Ni_16_Co_19_Mn_12_Cr_16_P_15_ alloy ingot after arc remelting on a copper plate, observed in the SEM at lower magnification. Within the eutectic colonies, a fine eutectic structure is visible. The EDS maps indicate an apparently uniform distribution of alloying elements (Fe, Ni, Co, Cr, Mn, P) in these regions, consistent with the resolution limits of the technique. At this magnification only minor inhomogeneities can be distinguished, whereas clearer chemical partitioning is observed in the inter-colony regions.

Figure 4 presents the eutectic microstructure at higher magnification. At this scale, the rod-like morphology becomes evident, with local lamellar segments. In the colony interiors, rods appear continuous and well-arranged. In the inter-colony regions, the microstructure is noticeably coarser and more irregular. EDS analysis shows that brighter areas are enriched in Fe, Ni, and Mn, with lower Co and a depletion in P and Cr, whereas darker areas are enriched in P and Cr, slightly richer in Co, and depleted in Fe, Ni, and Mn.

### 3.4. Differential Scanning Calorimetry (DSC) of the Ribbon

**Figure 5:**
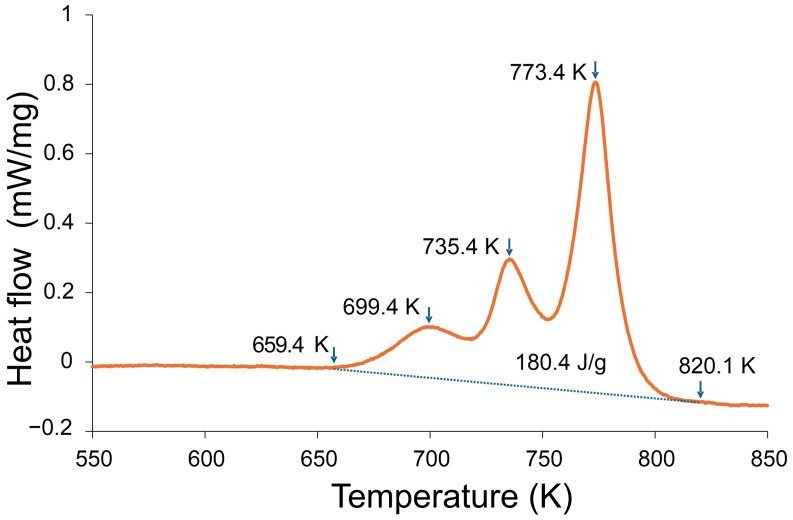
DSC curve of the amorphous ribbon of the Fe_22_Ni_16_Co_19_Mn_12_Cr_16_P_15_ alloy recorded at a heating rate of 20 K/min. Three exothermic events were detected, with the onset of crystallization at 659 K (~386 °C), subsequent peaks at 699 K (~426 °C) and 735–773 K (~462–500 °C), and completion near 820 K (~547 °C). The total crystallization enthalpy was 180.4 J/g.

This subsection analyzes the devitrification sequence of the amorphous ribbon (Figure 5), including the three exothermic events and their interpretation. 

Figure 5 shows the DSC curve of the amorphous ribbon of the Fe_22_Ni_16_Co_19_Mn_12_Cr_16_P_15_ alloy recorded at a heating rate of 20 K/min. Three distinct exothermic events are observed: the onset of crystallization at 659 K (~386 °C), a subsequent peak at 699 K (~426 °C), and a broader event in the range 735–773 K (~462–500 °C). The crystallization process is completed near 820 K (~547 °C). The total crystallization enthalpy amounts to 180.4 J/g, confirming the multistage character of devitrification in this alloy.

The DSC curve of the Fe_22_Ni_16_Co_19_Mn_12_Cr_16_P_15_ ribbon exhibits three exothermic peaks, indicating a multi-stage crystallization sequence. Similar behavior is well documented for transition metal–phosphorus (TM–P) metallic glasses, where crystallization proceeds through early TM-rich ordering, an intermediate stage associated with the development of a TM-rich phase or the onset of P-rich intermetallic formation, and a final high-enthalpy main crystallization event producing a TM solid solution together with P-rich phosphides [22,23,24,25]. The first low-enthalpy exotherm (≈699 K) corresponds to the onset of primary TM-rich ordering while P remains largely in the amorphous matrix. The second peak (≈735 K) is attributed to the further development of this TM-rich phase or early phosphide formation. The third and most intense exotherm (≈773 K) represents the principal crystallization stage producing a transition metal solid solution and phosphorus-rich phosphides. This interpretation is consistent with crystallization pathways reported for Fe–P, Ni–P and multicomponent TM–P metallic glass systems.

### 3.5. X-Ray Diffraction (XRD)

**Figure 6:**
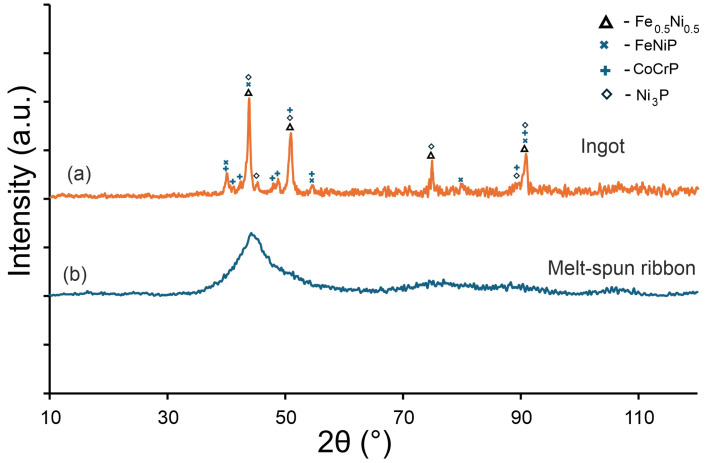
XRD patterns of the Fe_22_Ni_16_Co_19_Mn_12_Cr_16_P_15_ alloy: (**a**) arc-melted ingot, containing phases isomorphic with Fe–Ni (Fm-3 m), CrCoP (Pnma), Ni_3_P (I-4), and MnNiP (P-62 m), (**b**) melt-spun ribbon exhibiting a fully amorphous character.

This subsection presents the XRD patterns used to identify the phases in the arc-melted ingot and to confirm the fully amorphous character of the melt-spun ribbon (Figure 6).

Figure 6 shows the XRD patterns of the Fe_22_Ni_16_Co_19_Mn_12_Cr_16_P_15_ alloy obtained for the ingot and for the ribbon. In the ingot, solidified at a lower cooling rate, distinct crystalline reflections were detected. These peaks were assigned to phases isomorphic with the Fe–Ni solid solution (cubic, Fm-3 m), CrCoP-type (orthorhombic, Pnma), Ni_3_P-type (tetragonal, I-4), and MnNiP-type (hexagonal, P-62 m). In contrast, the melt-spun ribbon exhibits only a broad amorphous halo in the range of 2θ ≈ 40–50°, confirming its fully amorphous character and the absence of long-range crystalline order. The comparison in Figure 6 illustrates the difference between the fully amorphous melt-spun ribbon and the multiphase microstructure of the arc-melted ingot. The XRD pattern of the ingot is included because its crystalline phases were analyzed in detail by SEM/EDS and XRD, providing a reference for the phases formed under slow cooling.

## 4. Discussion

DSC analysis confirms the complex thermal behavior of the Fe_22_Ni_16_Co_19_Mn_12_Cr_16_P_15_ alloy. During heating, two distinct endothermic effects were detected, with the main one corresponding to melting. As established in the literature, first-order transitions are asymmetric: melting enthalpies exceed the corresponding crystallization enthalpies, and the processes are accompanied by hysteresis [26]. The same tendency is observed in this alloy –despite the strong eutectic transformation, the absolute enthalpy of crystallization remains lower than that of melting.

Upon cooling, DSC revealed a strong exothermic effect related to eutectic crystallization. This effect was considerably more pronounced than in the Fe_22_Ni_16_Co_19_Mn_12_Cr_16_P_15_ alloy reported previously [16], where in addition to the eutectic reaction, several effects related to primary and non-eutectic phase crystallization were recorded. In the present alloy, the eutectic reaction dominates, indicating that the compositional modification effectively increased its contribution at the expense of other transformations.

Thermographic measurements support these findings, showing a distinct temperature arrest (~1007 K) corresponding to eutectic crystallization. The simplicity of the thermographic curves contrasts with the multi-step character observed earlier in Cr_16_Mn_16_Fe_16_Co_16_Ni_16_P_20_, where successive arrests reflected the crystallization of several phases. In Fe_22_Ni_16_Co_19_Mn_12_Cr_16_P_15_, both DSC and thermography consistently demonstrate that crystallization proceeds predominantly through the eutectic reaction. These results clearly demonstrate that the compositional modification was successful, leading to a simplified sequence of phase transformations and a significantly increased eutectic fraction compared to the previously studied alloy.

The agreement between DSC and thermography further confirms the dominant role of the eutectic transformation in this alloy. Both methods consistently reveal a simplified sequence of transformations, without additional thermal effects that would indicate primary or non-eutectic crystallization. This coherence of thermal analyses strengthens the conclusion that the applied compositional modification effectively stabilized eutectic solidification.

The lower apparent arrest in thermography (~1007 K) reflects surface-dominated cooling (ROI, emissivity, convection), whereas DSC reports the bulk eutectic range (~1256–1281 K) under a controlled ramp. The lower apparent temperature in thermography results from the much higher cooling rate on the copper plate compared with the DSC experiment (20 K min^−1^), which causes significant undercooling prior to solidification. The plateau observed at ~1007 K therefore corresponds to eutectic crystallization under undercooled conditions rather than to the equilibrium solidus temperature.

The microstructural features observed in the Fe_22_Ni_16_Co_19_Mn_12_Cr_16_P_15_ alloy are consistent with general trends described for eutectic alloys. The classical work [27] on the Cu–Al–Ag system demonstrated that lamellar and rod-like morphologies can develop depending on solidification conditions. Similar transitions were reported in Sn–Ag alloys [28], where lamellar and rod-like eutectics coexisted in interdendritic regions. In the field of high-entropy alloys, the terminology lamellar or rod-like eutectic morphology has been explicitly adopted [29], and more recent studies classify the morphology unambiguously as rod-like whenever this type clearly dominates, even in the presence of local lamellar fragments [30].

The gray/bright regions visible in Figure 3a, acquired in BSE mode, are depleted in P and Cr but enriched in Fe, Ni, and Mn, corresponding to a Fe–Ni–Mn solid solution matrix (Fm-3 m). The darker P/Cr-rich regions can be assigned to transition metal phosphides, including CrCoP-type (Pnma), Ni_3_P-type (I-4), and MnNiP-type (P-62 m), consistent with the phases identified by XRD (Figure 6).

Closer analogies are provided by studies of multicomponent eutectics. Kakitani et al. [31], investigating the Al–33%Cu eutectic, showed that colony interiors are fine and ordered, while colony boundaries are coarser, irregular, and accompanied by segregation. Cai et al. [32], analyzing the Al–Cu–Si–Mg system, demonstrated coupled lamellar growth of α-Al/Al_2_Cu supplemented by a rod-like Q phase, with cell boundaries undergoing significant coarsening, growth decoupling, and compositional changes. Our observations fit well into this picture: the Fe_22_Ni_16_Co_19_Mn_12_Cr_16_P_15_ alloy shows colonies with relatively fine eutectic rods inside, but inter-colony regions are coarser and chemically heterogeneous. This partitioning favors the simultaneous presence of Fe–Ni solid solutions and transition metal phosphides, which is consistent with the multiphase constitution identified by XRD in the ingot.

Although EBSD can provide crystallographic phase identification, its applicability is limited in nanoscale or compositionally complex multiphase materials. Limited spatial resolution in EBSD causes the signal from very fine or closely intermixed phases to overlap, so the Kikuchi pattern does not originate from a single phase. Moreover, nanocrystalline or chemically heterogeneous regions often produce blurred or weak Kikuchi bands, which cannot be reliably matched to reference patterns. Consequently, most points cannot be indexed, making phase identification unreliable [33,34,35]. In the present alloy, the eutectic colonies contain nanoscale P-containing phosphides for which no validated EBSD reference data exist. Therefore, the BSE–EDS–XRD approach was used.

The results confirm that crystallization of the amorphous ribbon proceeds in a multistage manner. A comparable onset temperature has been reported for other P-containing alloys, such as Ni–P ribbons [5] and Pd–Ni–P glasses [13], both showing crystallization at ~650 K (~380 °C). In Fe–Ni–P–C ribbons, Małachowska et al. [6] observed a three-step crystallization sequence starting at ~392 °C and ending at ~470 °C, with a narrower crystallization window compared to the present alloy. Electroless Ni–P coatings typically crystallize at even lower temperatures, with Ni_3_P-related exotherms in the range 350–400 °C [12,36].

In contrast, the Fe_22_Ni_16_Co_19_Mn_12_Cr_16_P_15_ ribbon exhibits a broader crystallization interval (ΔTc ≈ 161 K), ending at ~547 °C, which demonstrates higher thermal stability compared to simpler Ni–P or Fe–Ni–P–C systems. The presence of three separate exothermic events is in line with the complex composition of the alloy, where phosphorus favors the glassy state during rapid quenching but promotes the formation of phosphides upon heating.

These XRD results are consistent with SEM/EDS observations, where brighter inter-colony areas were enriched in Fe, Ni, and Mn, and darker regions contained more P and Cr together with some Co. Such compositional partitioning explains the simultaneous formation of Fe–Ni solid solutions and transition metal phosphides revealed by diffraction. Similar segregation phenomena and precipitation of phosphides or borides have been documented in multicomponent alloys with non-metallic additions, further supporting the interpretation of the present findings.

The presence of as many as four crystalline phases in the ingot reflects the chemical complexity of the alloy and the role of phosphorus in stabilizing transition metal phosphides. These findings agree with SEM/EDS observations, which revealed local compositional differences in the inter-colony regions: brighter areas were enriched in Fe, Ni, and Mn, whereas darker regions contained more P and Cr together with some Co. Such partitioning promotes the simultaneous formation of Fe–Ni solid solutions and various phosphides, in line with the multiphase assemblage identified by XRD.

The presence of multiple crystalline phases in the ingot confirms that chemical partitioning occurs during slow cooling, particularly because phosphorus promotes the formation of transition metal phosphides under near-equilibrium conditions. On the basis of the present observations, optimization should aim to suppress segregation by increasing the effective cooling rate. Previous studies have shown that modified arc-melting configurations enabling directional heat extraction, such as pseudo-float melting in a ladle-type arc-melt furnace [37] or multi-electrode arc-melting systems designed to improve thermal homogeneity [38], can enhance amorphization in alloys with moderate glass-forming ability. While these processing routes were not evaluated in this study, they represent potential directions for future optimization of P-containing HEA-type alloys.

Similar phenomena have been reported in other multicomponent alloys containing phosphorus, boron, or silicon. In Ni- and Fe-based alloys, P segregation promotes the precipitation of phosphides at grain boundaries [2], while in HEAs with B or Si additions, stable borides or silicides form under comparable conditions [4]. These results confirm that the coexistence of solid solutions and intermetallic compounds is a typical feature of high-entropy alloys with non-metallic elements.

For the melt-spun ribbon, the absence of crystalline reflections demonstrates that rapid cooling suppressed elemental segregation and phosphide formation, enabling the preservation of the amorphous state. This outcome is consistent with DSC results, which revealed multiple exothermic effects corresponding to crystallization events upon heating. Together, the XRD and DSC findings confirm that while slow cooling leads to the formation of several stable crystalline phases, rapid cooling is sufficient to retain a fully amorphous structure.

No glass transition (Tg) was detected in the DSC curve of the Fe_22_Ni_16_Co_19_Mn_12_Cr_16_P_15_ ribbon, and the γ parameter cannot therefore be evaluated. As an experimentally accessible thermal characteristic, we consider the observable crystallization span. The ribbon begins to crystallize at approximately 659 K, and the multistage crystallization sequence extends up to ~820 K. This upper crystallization temperature is higher than in several well-documented transition metal–phosphorus glass-forming alloys: Ni–P and Pd–Ni–P glasses normally complete crystallization below ~720–780 K [5,13], Fe–Ni–P–C ribbons crystallize up to ~740–760 K [6], and Ni–P coatings show even lower crystallization temperatures [12,36]. Alloys from the Ni–Pd–P–B family with high glass-forming ability also complete crystallization below ~780 K [39]. Although the T_x_/T_l_ ratio of the present alloy (~0.53) is moderate, the comparatively high final crystallization temperature indicates enhanced thermal resistance of the amorphous state relative to most TM–P systems and highlights the interest in HEA-type compositions with controlled chemical complexity and elevated P content.

Although the present study does not address mechanical properties, it should be emphasized that its primary objective was to clarify the thermal stability, solidification behavior and crystallization pathway of the Fe_22_Ni_16_Co_19_Mn_12_Cr_16_P_15_ alloy. These characteristics determine whether an alloy is capable of retaining an amorphous structure under rapid cooling and therefore form the basis for future evaluation of its mechanical performance. The enhanced thermal resistance of the amorphous state, the comparatively high final crystallization temperature and the simplified transformation sequence revealed in this work indicate that the alloy is a promising candidate for subsequent studies, including systematic mechanical testing once fully amorphous bulk samples are available. The present results thus provide a necessary foundation for such follow-up investigations while remaining within the defined thermal and structural scope of this study.

## 5. Conclusions

Thermographic analysis of the Fe_22_Ni_16_Co_19_Mn_12_Cr_16_P_15_ ingot revealed a distinct temperature arrest at ~1007 K, corresponding to eutectic crystallization. The transformation was accompanied by macroscopic contraction manifested as a flattened surface region, providing direct evidence of the eutectic reaction during rapid cooling.

DSC measurements confirmed that solidification of the ingot is dominated by the eutectic reaction, whose enthalpy (−170.7 J/g) was significantly stronger than the thermal effects associated with primary or non-eutectic phases. Compared with the previously studied Cr_16_Mn_16_Fe_16_Co_16_Ni_16_P_20_ alloy, the present composition exhibits a simplified transformation sequence and an increased eutectic fraction.

This behavior arises from the targeted compositional modification: increasing Fe and Co while reducing Mn decreases the tendency for primary solid solution crystallization, and lowering the P content from 20 to 15 at.% limits the formation of high-temperature phosphides. These changes shift the alloy closer to a cooperative eutectic-growth regime, thereby suppressing multiple competing reactions and stabilizing a simpler solidification pathway dominated by the eutectic transformation.

DSC analysis of the melt-spun ribbon showed a three-stage crystallization process (onsets at 659 K, 699 K, and 735–773 K, completion near ~820 K) with a total enthalpy of 180.4 J/g. The crystallization span is wider than in simpler Ni–P or Fe–Ni–P–C alloys, indicating enhanced thermal stability of the amorphous phase. The comparatively high final crystallization temperature further demonstrates an increased resistance of the amorphous structure to thermally activated decomposition relative to many transition metal–phosphorus systems.

SEM/EDS observations revealed that the microstructure of the ingot consists of eutectic colonies with predominantly rod-like morphology, while inter-colony regions show pronounced chemical partitioning: the brighter areas enriched in Fe, Ni and Mn correspond to a Fe–Ni–Mn solid solution matrix, whereas darker regions enriched in P and Cr correspond to transition metal phosphides.

XRD confirmed the presence of four crystalline phases isomorphic with Fe–Ni (Fm-3 m), CrCoP (Pnma), Ni_3_P (I-4), and MnNiP (P-62 m) in the ingot, while melt-spun ribbons exhibit a fully amorphous structure. Together, the DSC, SEM/EDS and XRD results demonstrate that rapid cooling suppresses segregation and phosphide formation, enabling complete amorphization, whereas slower cooling leads to a multiphase crystalline structure.

Overall, the combined results show that the Fe_22_Ni_16_Co_19_Mn_12_Cr_16_P_15_ alloy possesses good glass-forming ability under rapid cooling and that the simplified, eutectic-dominated transformation pathway is a direct consequence of the controlled adjustment of Fe/Co/Mn ratios and phosphorus content. Under near-equilibrium conditions, however, phosphorus strongly promotes phosphide formation, and therefore, the precise control of cooling rates is essential for retaining the amorphous state and avoiding complex multiphase crystallization. These mechanistic insights provide a basis for further optimization of P-containing HEAs and motivate future work aimed at evaluating the mechanical performance of fully amorphous bulk samples derived from this alloy.

## Data Availability

The original contributions presented in this study are included in the article. Further inquiries can be directed to the corresponding author.
